# Genomic and Functional Analyses of the 2-Aminophenol Catabolic Pathway and Partial Conversion of Its Substrate into Picolinic Acid in *Burkholderia xenovorans* LB400

**DOI:** 10.1371/journal.pone.0075746

**Published:** 2013-10-04

**Authors:** Bernardita Chirino, Erwin Strahsburger, Loreine Agulló, Myriam González, Michael Seeger

**Affiliations:** Laboratorio de Microbiología Molecular y Biotecnología Ambiental, Departamento de Química and Center for Nanotechnology and Systems Biology, Universidad Técnica Federico Santa María, Valparaíso, Chile; Auburn University, United States of America

## Abstract

2-aminophenol (2-AP) is a toxic nitrogen-containing aromatic pollutant. *Burkholderia xenovorans* LB400 possess an *amn* gene cluster that encodes the 2-AP catabolic pathway. In this report, the functionality of the 2-aminophenol pathway of *B. xenovorans* strain LB400 was analyzed. The *amnRJBACDFEHG* cluster located at chromosome 1 encodes the enzymes for the degradation of 2-aminophenol. The absence of *habA* and *habB* genes in LB400 genome correlates with its no growth on nitrobenzene. RT-PCR analyses in strain LB400 showed the co-expression of *amnJB*, *amnBAC, amnACD*, *amnDFE* and *amnEHG* genes, suggesting that the *amn* cluster is an operon. RT-qPCR showed that the *amnB* gene expression was highly induced by 2-AP, whereas a basal constitutive expression was observed in glucose, indicating that these *amn* genes are regulated. We propose that the predicted MarR-type transcriptional regulator encoded by the *amnR* gene acts as repressor of the *amn* gene cluster using a MarR-type regulatory binding sequence. This report showed that LB400 resting cells degrade completely 2-AP. The *amn* gene cluster from strain LB400 is highly identical to the *amn* gene cluster from *P. knackmussi* strain B13, which could not grow on 2-AP. However, we demonstrate that *B. xenovorans* LB400 is able to grow using 2-AP as sole nitrogen source and glucose as sole carbon source. An *amnBA* ^−^ mutant of strain LB400 was unable to grow with 2-AP as nitrogen source and glucose as carbon source and to degrade 2-AP. This study showed that during LB400 growth on 2-AP this substrate was partially converted into picolinic acid (PA), a well-known antibiotic. The addition of PA at lag or mid-exponential phase inhibited LB400 growth. The MIC of PA for strain LB400 is 2 mM. Overall, these results demonstrate that *B. xenovorans* strain LB400 posses a functional 2-AP catabolic central pathway, which could lead to the production of picolinic acid.

## Introduction

Several aromatic compounds with nitro and amino groups such as 2-aminophenol (2-AP) are toxic and persistent organic pollutants (POPs) in the environment. 2-AP is a compound used in the production of dyes, plastics and pharmaceuticals, which could be released in industrial wastewaters, polluting the environment [1], [2]. 2-AP is cytotoxic [3] and has carcinogenic potential [4]. Therefore, the removal of 2-AP is required for the clean-up of polluted sites. Some microorganisms are able to degrade aerobically 2-AP [1], [5].

**Figure 1:**
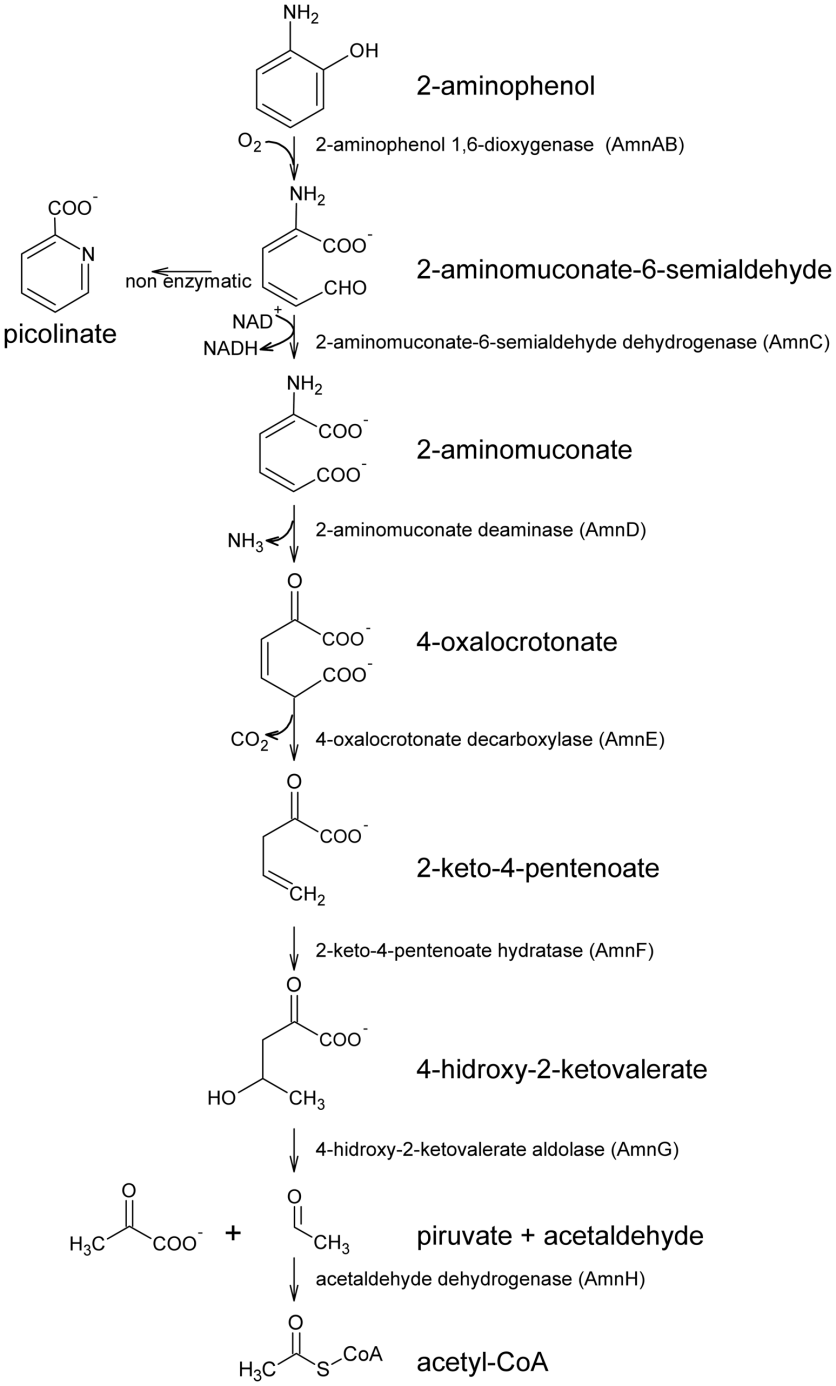
Model of the aerobic metabolism of 2-aminophenol in bacteria. The substrate and product(s) of each enzyme are indicated. The non-enzymatic production of picolinic acid during 2-AP catabolism in *B. xenovorans* LB400 is depicted.

The aerobic 2-AP metabolic pathway (Fig. 1) has been described in *Pseudomonas pseudoalcaligenes* JS45, *Pseudomonas putida* HS12, *Pseudomonas sp*. AP-3 and *Pseudomonas knackmussii* B13 [6]–[9]. The enzymes AmnBA, AmnC, AmnE, AmnD, AmnF, AmnG, AmnH are involved in the degradation of 2-AP into pyruvate and acetyl-CoA [1], [8], [10]–[12]. 2-aminophenol-1,6-dioxygenase (AmnAB) converts 2-AP through an extradiol meta cleavage into 2-aminomuconic 6-semialdehyde (2-AMS). 2-AMS is further oxidized by the AmnC dehydrogenase into 2-aminomuconic acid (2-AM), which is converted by AmnD into 4-oxalocrotonic acid with a concomitant release of ammonium. 4-oxalocrotonic acid is further degraded by the enzymes AmnE, AmnF, AmnG and AmnH into pyruvate and acetyl-CoA.

*B. xenovorans* LB400 is a model bacterium for the degradation of polychlorobiphenyls (PCBs) and other aromatic compounds [13]–[15]. Its genome comprises a major chromosome (C1), a minor chromosome (C2) and one megaplasmid (MP) [15]. A wide range of aromatic compounds including PCBs, hydroxyphenylacetates and hydroxybenzoates are metabolized by a number of peripheral catabolic pathways into eleven central catabolic pathways [14]–[17]. Strain LB400 possesses the *amn* genes that encode the enzymes from the 2-AP central catabolic pathway (Fig. 1), but the functionality of this pathway has not been studied. The aim of this study was to determine the functionality of the 2-AP catabolic pathway from *B. xenovorans* LB400.

## Materials and Methods

### Chemicals

2-Aminophenol (99% purity), nitrobenzene (>98% purity) and picolinic acid (99% purity), were obtained from Sigma-Aldrich (Saint Louis, MO, USA).

### Bacterial Strain and Culture Conditions

*B. xenovorans* LB400 was cultivated in Luria Bertani (LB) medium and in nitrogen-free BLKN medium [18] using glucose (10 mM) as sole carbon source at 30°C. For selective growth of strain LB400, OF basal medium agar (Oxoid) with glucose 1% (w/v) and supplemented with bacitracin (0.2 U/mL) and polymyxin B (0.3 U/mL), and BLKN minimal medium agar with NH_4_Cl (1 mM) and crystal of biphenyl (1 mg) were used.

To study growth using 2-AP as carbon and nitrogen source in liquid media, LB400 cells previously cultured in BLKN medium using biphenyl (10 mM) as sole carbon source and NH_4_Cl (10 mM) as sole nitrogen source, harvested by centrifugation and washed twice with sodium phosphate buffer (0.02 M, pH 7.0), were incubated in BLKN minimal medium with benzoate (10 mM) and NH_4_Cl (10 mM) for 24 h at 30°C on a rotary shaker (150 rpm). The cells were harvested by centrifugation, washed twice with sodium phosphate buffer and suspended in BLKN medium supplemented with glucose (10 mM), 2-AP (1 mM) or NH_4_Cl (1 mM) in darkness. Growth was determined by measuring turbidity at 525 nm and by counting colony-forming units (CFU). The CFU were determined using a microdot method in LB plates and were calculated as the mean ± SD of at least three independent experiments. To study the growth of strain LB400 using 2-AP as nitrogen and carbon source on solid media, BLKN agar with and without glucose (10 mM) was used. 2-AP crystals (1 mg) and/or an aliquot (5 µL) of nitrobenzene (1 M) were added. The plates were inoculated with LB400 cells and incubated at 30°C for 48 h.

Minimum inhibitory concentration (MIC) of PA for strain LB400 was determined by Mueller-Hinton broth (Difco, Detroit, USA) microdilution at 5×10^5^ CFU/mL according to Clinical and Laboratory Standards Institute (CLSI) guidelines [19]. Mueller-Hinton medium was supplemented with PA in the concentration range from 0.032 mM (4 µg/ml) to 4.159 mM (512 µg/ml): 0.032, 0.065, 0.130, 0.260, 0.520, 1.040, 2.079 and 4.159 mM. MIC for PA was the lowest concentration at which no LB400 growth was observed. MIC analysis was done in triplicate.

### Generation of an *amnBA* ^−^ Mutant Strain by Gene Disruption

A *B. xenovorans* LB400 mutant defective for 2-aminophenol 1,6-dioxygenase encoded by the *amnB* and *amnA* genes was constructed by gene disruption through homologous recombination. For this purpose, an *amnBA* fragment (430 bp), which was amplified using the primers AR1 y BF2, was cloned in the pCR2.1-TOPO cloning vector. Resulting plasmid pCR2.1amnBA was digested with EcoRI, and the EcoRI fragment of pCR2.1amnBA was cloned into EcoRI site of pK18mobsacB and introduced into *E. coli* CC118 [λpir] prior to selection for Km-resistance clones carrying *amnBA* fragment. Resulting plasmid pK18mobsacBamnBA was transferred into *B. xenovorans* LB400 by triparental mating with *E. coli* HBH101 (pRK600) as helper strain [20]. Km-resistance colonies able to utilize biphenyl as sole carbon source were selected. The *amnBA* ^−^ mutant was verified by PCR amplification with primer sets M13R and AR1, M13R and CR, M13R and DR, JF and CR.

### RNA Isolation

Total RNA was isolated from LB400 cells using an RNeasy mini kit (Qiagen, Hilden, Germany) according to the manufacturers’ recommendations. DNase I treatment was carried out using the RNase-Free DNase set (Qiagen, Hilden, Germany) to degrade any residual DNA. Amplification of 16S rRNA gene and *amnB* gene using specific primer sets (Table 1) were used as controls to exclude DNA contamination. The RNA concentration was quantified using a Qubit fluorometer (Invitrogen) and a Nanodrop spectrophotometer (Thermo Scientific). RNA integrity was tested by agarose (1%) gel electrophoresis.

**Table 1:** Primers used in this study.

Gene	Primer	Sequence (5′–3′)		Reference
*amnR*	RF	ACCTACCGCTACATACGACTG		This study (*)
	RR	TCAAACCACTGCTGCTCATC		*
*amnJ*	JF	CTCGGATGTCTATGTCTCGG		*
*amnA*	AF1	GTGGTGGCGCTATTGTCCAG		*
	AR1	CAGCAAGTGCTTTGCCGGCT		
*amnB*	BF1	CGCATCTGGTCTATGGGGA		*
	BF2	CTACGACGGCTACTTGTGGGA		*
	BR1	GTTGCCAGTGCCGATGAC		*
	BR2	GACCTCGGTTCGTTCTGA		*
*amnC*	CR	GACAGCCTCATCAACCATCTC		*
*amnD*	DF	TTTTTGTCTCAGGCACCAG		*
	DR	ATCGGGTCAGGCAGCTGA		*
*amnE*	EF	AGGTCGGCTCCACGGCGGC		*
	ER	GATGACATCGGACAGCCCCAT		*
*amnG*	GR	CCTGAGGCACAGAGTATTGATGGC		*
16S rRNA	27f	AGAGTTTGATCMTGGCTCAG		16
	1492r	TACGGYTACCTTGTTACGACT		16
*ftsZ*	FZF1		CGATTACGGTGCGCTGGATA	*
	FZR1		ATGCCGGAATGTCGTACGTG	*
internal sequence (pK18mobsacB)	M13R		AACAGCTATGACCATG	Life Technologies

### RT-PCR and Real-Time RT-PCR

Reverse transcription-PCR (RT-PCR) was carried out with sequence-specific primers design in this study (Table 1) by using SuperScript One-step RT-PCR with Platinum Taq (Invitrogen, Carlsbad, USA). The co-expression assays were performed using the primers indicated in Table 1. The cycles of amplification (35 cycles) was carried outs as follows: 95°C for 1 min, 60°C for 0.5 min, 72°C for 2 min, after an initial denaturation at 95°C for 5 min. Negative and positive controls were included in each RT-PCR assay. At least two independent RT-PCR reactions for each condition were done to assess reproducibility.

For RT-qPCR, total RNA (100 ng) was transcribed with Verso cDNA kit (Thermo Scientific, Lafayette, USA). The RT-qPCR was performed according to the MIQE guidelines [21]. Real-time PCR was performed on Mx3000P qPCR system (Stratagene), using the Kapa Sybr fast qPCR master mix (2×) universal kit (Kapabiosystems, Boston, USA) and 0.3 µM of each primer. Samples were initially denatured at 95°C for 5 min. A 40-cycle amplification and quantification protocol (95°C for 15 s, 62°C for 15 s and 72°C for 15 s) with a single fluorescence measurement per cycle followed by a melting-curve program (95°C for 15 s, 25°C for 0.1 s, 70°C for 15 s and 95°C for 0.1 s) were used according to manufacturer’s recommendations. The PCR melting curves confirm the amplification of a single product for each primer pair. The BF2 and BR1 primers were used for *amnB* gene amplification, yielding a product of 208 bp. The *ftsZ* (BxeA0491) gene was amplified as a reference gene, yielding an amplicon of 113 bp. A standard curve in triplicate was made with 5 serial dilutions (10 fold) for each amplicon in a linear range (9.2 ng –0.09 pg) of LB400 genomic DNA. qPCR efficiencies were calculated from the slopes of the log-linear portion of the calibration curves, using the equation E = 10^(−1/slope)^. The qPCR efficiency rate was 1.95 for both *amnB* and *ftsZ* genes. The *ftsZ* gene was stably expressed according to the algorithms of BestKeeper [22]. Relative *amnB* gene expression ratios were determined by the Pfaffl method [23], normalizing the gene expression to LB400 cells grown in BLKN medium with glucose as sole carbon source and NH_4_Cl as sole nitrogen source.

### Degradation Assay

Resting cells (turbidity _525 nm_ = 0.6) were incubated in sodium phosphate buffer (50 mM, pH 7.2) with 2-AP (1 mM). Aliquots of cell suspensions were taken at different incubation times and centrifuged (19,283×g for 2 min). Assays with boiled cells and without cells were used as controls. Cell-free supernatants were analyzed, using a Waters liquid chromatograph model 515 equipped with a UV detector and a RP-C18/Lichrospher 5-mm column (Supelco, Bellefonte, USA). The aqueous mobile phase contained 20% methanol, 10% acetonitrile and 70% sodium acetate (0.1 M) solution. The flow rate was 1 mL min^−1^. 2-AP was quantified using calibration curves with authentic standards. Resting cells experiments were performed in triplicate.

### Metabolism of 2-AP

LB400 cells grown in BLKN medium were incubated in presence of 2-AP (1 mM) in darkness at 30°C. Aliquots (2 mL) were collected by centrifuged (19,283×g for 2 min) at different times and cell-free supernatants were analyzed by reverse phase chromatography with a Jasco liquid chromatograph equipped with a diode array detector and a Whatman C-18 column (25 cm by 4.6 mm ID). The aqueous mobile phase contained 20% methanol, 20% acetonitrile and 0.1% phosphoric acid and the flow rate was 1.0 mL min^−1^. The compounds were monitored at 278 nm. Under these conditions, 2-AP and picolinic acid have retention times of 4.56 and 3.73 min, respectively. 2-AP and picolinic acid were quantified using commercial authentic standards.

### Bioinformatics Analysis

The *amn* genes and the neighborhood in the genome of strain LB400 was analyzed using the Vector NTI suite 9.0 software. For the sequence alignment the database of the National Center for Biotechnology Information (NCBI/BLAST Home) (http://blast.ncbi.nlm.nih.gov/Blast.cgi) was used. Clustal W software was used for analysis of the sequence of *amnB* gene with selected sequences retrieved from GenBank. The software package MEGA 5.0 [24] was used for phylogenetic analyses. A phylogenetic tree was built by using the Neighbor-Joining method with a bootstrap analysis of 5000.

## Results

### Bioinformatic Analysis of the *amn* Genes in *B. xenovorans* Strain LB400

**Figure 2:**
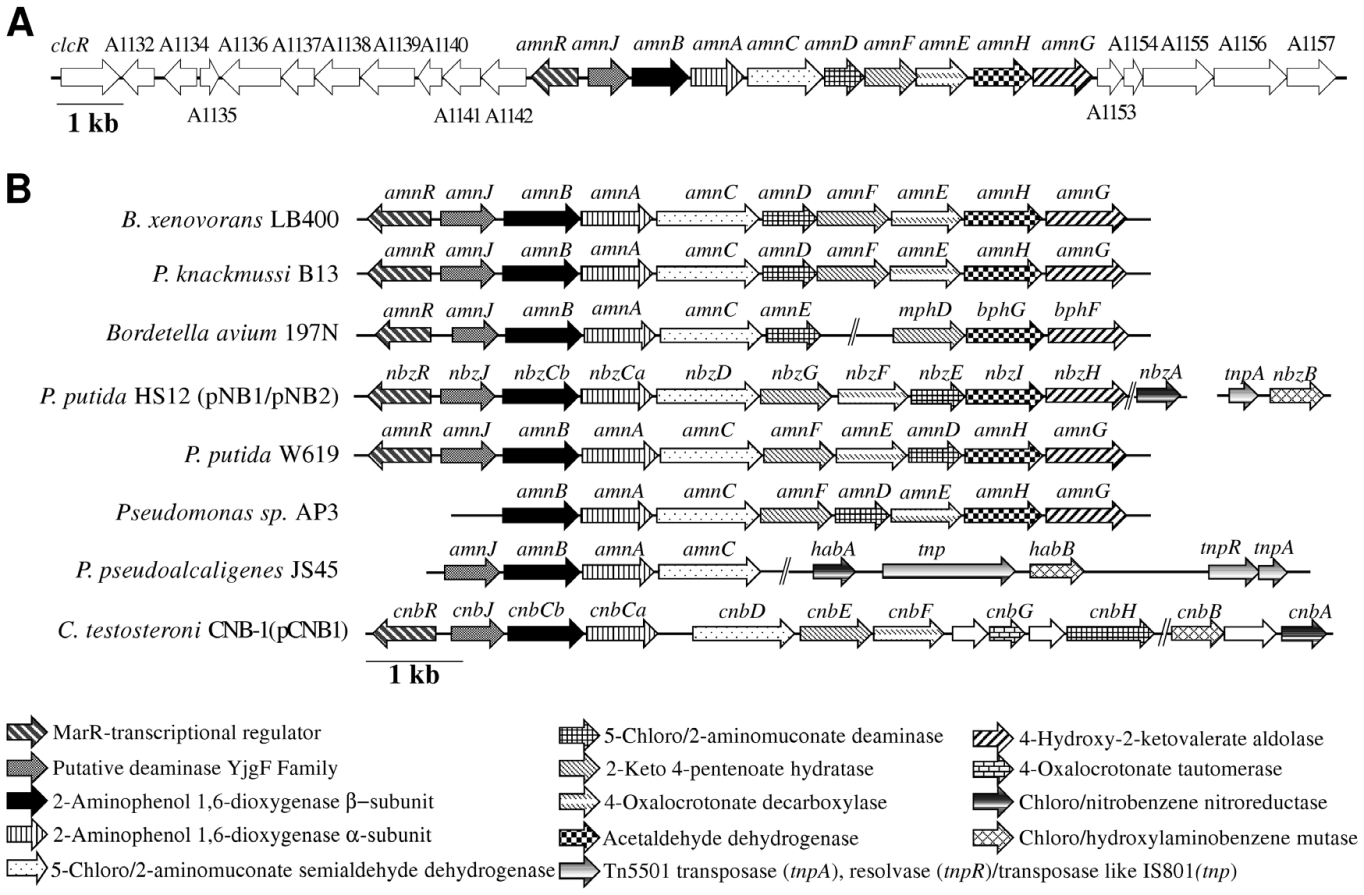
Organization of the predicted genes encoding the 2-aminophenol central pathway in *B. xenovorans* LB400 and comparison of bacterial *amn* gene clusters. A, The *amn* genes encoding the 2-aminophenol central pathway located at the major chromosome (C1). Genes of the *amn* gene cluster neighborhood are represented with white arrows. The genes located upstream of the *amn* gene cluster encode the following proteins: *clcR*, LysR-type transcriptional regulator of the 3-chlorocatechol pathway; BxeA1132, pseudogene; BxeA1134, LysR-type transcriptional regulator; BxeA1135, XRE-type transcriptional regulator; BxeA1136 LysR-type transcriptional regulator; BxeA1137, MarR-type transcriptional regulator; BxeA1138, TetR-type transcriptional regulator; BxeA1139, hypothetical protein; BxeA1140, DoxX family protein; BxeA1141, TetR-type transcriptional regulator; BxeA1142, DoxX family protein. The genes located downstream of *amn* gene cluster encode the following proteins: BxeA1153, putative esterase; BxeA1154, TraG conjugal transfer coupling protein; BxeA1155, outer membrane protein from MFS multidrug efflux system; BxeA1156, inner membrane protein from MFS multidrug efflux system; BxeA1157, transmembrane protein from MFS multidrug efflux system. The sizes of the genes and the intergenic regions are on scale. B, Comparison of bacterial *amn* gene clusters. The *amn* sequences (accession number) are: *B. xenovorans* LB400 (NC007951), *P. knackmussii* B13 (AJ617740), *Bordetella avium* 197N (NC010645), *P. putida* HS12 plasmid pNB1 (AF319593) and plasmid pNB2 (AF319592), *P. putida* W619 (CP000949), *Pseudomonas* sp. AP3 (AB020521), *P. pseudoalcaligenes* JS45 (PPU93363), *Comamonas testosteroni* CNB-1 plasmid pCNB1 (EF079106).

The *amn* genes from *B. xenovorans* LB400 are clustered at C1 (Fig. 2). The *amn* gene cluster includes the BxeA1143, BxeA1144, BxeA1145, BxeA1146, BxeA1147, BxeA1148, BxeA1149, BxeA1150, BxeA1151 and BxeA1152 genes (hereafter *amnR, amnJ, amnB, amnA, amnC, amnD, amnF, amnE, amnH and amnG* genes, respectively) and has an extension of 8,529 bp. The predicted *amn* genes of strain LB400 are listed in Table 2. The *amnRJBACDFEHG* gene cluster encodes the enzymes of the catabolic pathway for the conversion of 2-aminophenol into pyruvate and acetyl-CoA (Fig. 1). The *amnBA* genes encode the 2-aminophenol-1,6-dioxygenase that open the 2-aminophenol ring via meta-cleavage, producing 2-AMS. The predicted *amnC* gene encodes 2-aminomuconic-6-semialdehyde dehydrogenase that oxidizes 2-AMS into 2-AM. The *amnD* gene encodes the enzyme 2-aminomuconic deaminase that converts 2-AM into 4-oxalocrotonic acid and ammonium, which could be used as nitrogen source for bacterial growth. The predicted *amnE* encodes the 4-oxalocrotonate decarboxylase, which converts 4-oxalocrotonate into 2-keto-4-pentenoate. The *amnF* gene encodes 2-keto-4-pentenoate hydratase that oxidizes 2-keto-4-pentenoate into 4-hidroxy-2-ketopentanoate. The predicted *amnG* gene encodes 4-hidroxy-2-ketovalerate aldolase that degrades 4-hidroxy-2-ketopentanoate into pyruvate and acetaldehyde. The *amnH* gene encodes the enzyme acetaldehyde dehydrogenase for the final conversion of acetaldehyde into acetyl-CoA. The *amnR* and *amnJ* genes are also present in the *amn* gene cluster from strain LB400. The *amnR* gene encodes a MarR-type transcriptional regulator, which probably acts as transcriptional repressor of the *amn* gene cluster. The *amnJ* gene is present in diverse bacterial *amn* gene clusters, suggesting that its product is required for the 2-AP biochemical pathway. Gaillard *et al*. [9] proposed that AmnJ is a ferredoxin-like protein. We have observed that the AmnJ protein has a conserved domain from the YjgF super family proteins that have enamine/imine deaminase activity [25]–[26].

**Table 2:** Identification of genes involved in the 2-aminophenol degradation pathway in *B. xenovorans* LB400.

ORF	Gene	Orientation	Size (aa)	Function	Homology^a^ (source)	Accession number	E value^b^	Amino acid identity	
								%	aa range
BxeA1143	*amnR*	R	205	MarR-family transcriptional regulator	MarR-family transcriptional regulator(*Bordetella avium* 197N)	ABE31805.1	1.0 E-110	79	154
BxeA1144	*amnJ*	F	137	Putative imino/enamina deaminase	Putative ferredoxin (*Pseudomonas putida* pNB1)	ABE31804.1	1.0 E-27	49	136
BxeA1145	*amnB*	F	304	2-Aminophenol 1,6-dioxygenase, β subunit	2-Aminophenol 1,6-dioxygenase β subunit(*Bordetella avium* 197N)	ABE31803.1	1.0 E-162	89	300
BxeA1146	*amnA*	F	270	2-Aminophenol 1,6-dioxygenase, α subunit	2-Aminophenol 1,6-dioxygenase α subunit(*Bordetella avium* 197N)	ABE31802.1	1.0 E-119	74	202
BxeA1147	*amnC*	F	492	2-Aminomuconate-6-semialdehyde dehydrogenase	2-Aminomuconate-6-semialdehyde dehydrogenase(*Bordetella avium* 197N)	ABE31801.1	0.0	88	434
BxeA1148	*amnD*	F	145	2-Aminomuconate deaminase	2-Aminomuconate deaminase(*Bordetella avium* 197N)	ABE31800.1	1.0 E-62	83	121
BxeA1149	*amnF*	F	269	2-Keto-4-pentenoate hydratase	2-Keto 4-pentenoate hydratase(*Alcaligenes sp*. O-1)	ABE31799.1	7.0 E-95	63	166
BxeA1150	*amnE*	F	254	4-Oxalocrotonate decarboxylase	4-Oxalocrotonate decarboxylase(*Burkholderia multivorans* CGD2M)	ABE31798.1	6.0 E-94	71	174
BxeA1151	*amnH*	F	313	Acetaldehyde dehydrogenase	Acetaldehyde dehydrogenase (acylating)(*Comamonas testosteroni CNB1* pCNB1)	ABE31797.1	1.0 E-143	83	262
BxeA1152	*amnG*	F	345	4-Hydroxy 2- ketovalerate aldolase	4-Hydroxy-2-ketovalerate aldolase(*Comamonas testosteroni CNB1* pCNB1)	ABE31796.1	1.0 E-164	87	290

a The *amn* genes from *B. xenovorans* LB400 and *P. knackmussii* B13 are almost identical; therefore the identity between them are not listed.

b E values are based on BLASTP results of the non-redundant NCBI database.

The genetic neighborhood of the *amn* gene cluster in strain LB400 was analyzed. The genes located upstream (between BxeA1142 and BxeA1131 (*clcR*) genes) and downstream (between BxeA1153 and BxeA1157) of the *amn* gene cluster were studied (Fig. 2A). Diverse transcriptional regulator genes are located upstream of the *amn* gene cluster. The BxeA1141, BxeA1138, BxeA1137, BxeA1136, BxeA1135 and BxeA1134 genes encode a TetR-type transcriptional regulator closely related to a TetR-type regulator from *Rhizobium leguminosarum* bv. viciae USDA 2370 (accession number EJB01162.1; 56% identity), a TetR-type transcriptional regulator closely related to a TetR-type regulator protein from *Acidovorax* sp. JS42 (ABM43064.1; 93% identity), a MarR-type transcriptional regulator closely related to a MarR-type regulator protein from *Acidovorax* sp. JS42 (ABM43065.1; 77% identity), a LysR-type transcriptional regulator closely related to a LysR-type regulator protein from *Bordetella petrii* DSM 12804 (CAP41606.1; 81% identity), a XRE-type transcriptional regulator closely related to a XRE regulator protein from *Alcaligenes* sp. HPC1271 (EKU29625.1; 86% identity), and a LysR-type transcriptional regulator closely related to a LysR-type regulator protein from *Acidovorax delafieldii* 2AN (EER60975.1; 67% identity), respectively (Fig. 2). Three genes located downstream of the *amn* gene cluster encode membrane transporter proteins. The BxeA1155, BxeA1156 and BxeA1157 genes encode three components (an outer membrane protein, an inner membrane protein and a transmembrane protein, respectively) of a major facilitator superfamily (MFS) multidrug efflux system. This multidrug efflux system possesses high identity with a multidrug efflux transporter pump from *Ralstonia solanacearum* GMI1000 [27], including the channel-forming component (CAD17593.1; 54% identity), the inner membrane protein (CAD17592.1; 58% identity) and the transmembrane protein (CAD17591.1; 57% identity), respectively. The BxeA1154 gene encodes a TraG conjugal transfer coupling protein that has 33% identity with the TraG protein (accession number AFT71063.1) from *Alcanivorax dieselolei* B5 [28].

The analysis of the genetic organization of the *amn* genes in bacteria indicate a conserved *amnJBACD* gene cluster encoding enzymes for the conversion of 2-AP until 4-oxalocrotonate, and *amnFEHG* genes encoding enzymes involved in the transformation of 4-oxalocrotonate into acetyl-CoA. Regulation of the *amn* gene cluster is generally mediated by an *amnR* gene-encoded Mar-type family transcriptional regulator located upstream of the *amn* gene cluster. *B. xenovorans* LB400, *P. knackmussii* B13 [9] and *Bordetella avium* 197N [29] shared the *amnRJBACD* gene organization (Fig. 2). *P. putida* strains HS12 and W619 shared the *amnRJBAC* gene cluster, whereas the *amnD* gene is located after the *amnFE* genes. *P. pseudoalcaligenes* strain JS45 and *Pseudomonas* sp. strain AP-3 showed a similar *amnBAC* gene organization, but in these strains the *amnR* gene and *amnRJ* genes have not been reported (Fig. 2). In *Pseudomonas* sp. strain AP-3 the *amnD* gene is located between *amnF* and *amnE* genes. *Comamonas testosteroni* strain CNB-1 possesses a similar gene cluster for the 2-amino-5-chlorophenol catabolism [30]. The *cnbJBACD* gene cluster that encodes the enzymes for the conversion of 2-amino-5-cholorophenol into 4-oxalocrotonate has the same gene organization (Fig. 2). The genetic organization of *amnFEHG* genes, which encode the enzymes for the conversion of 4-oxolocrotonate into acetyl-CoA and is conserved in other bacteria, is located in *B. xenovorans* strain LB400 downstream of the *amnRJBACD* gene cluster. However, in *Pseudomonas* strains HS12, W619 and AP-3, the *amnD* gene is located within the *amnFEGH* cluster (Fig. 2). The *amnFEGH* region has not been reported in the *amn* gene cluster of *P. pseudoalcaligenes* strain JS45.

The nitrobenzene catabolic peripheral pathway funnels into the 2-aminophenol central pathway in bacteria. The *habA* and *habB* genes that encode enzymes involved in the conversion of nitrobenzene into 2-aminophenol are absent in the genome of *B. xenovorans* LB400. In the 2-AP-degraders *P. putida* HS12 and *P. pseudoalcaligenes* JS45 the *hab* genes are located distantly from the *amn* gene cluster (Fig. 2). In accordance with the absence of the *hab* genes, strain LB400 was unable to grow using nitrobenzene as sole nitrogen or carbon source.

**Figure 3:**
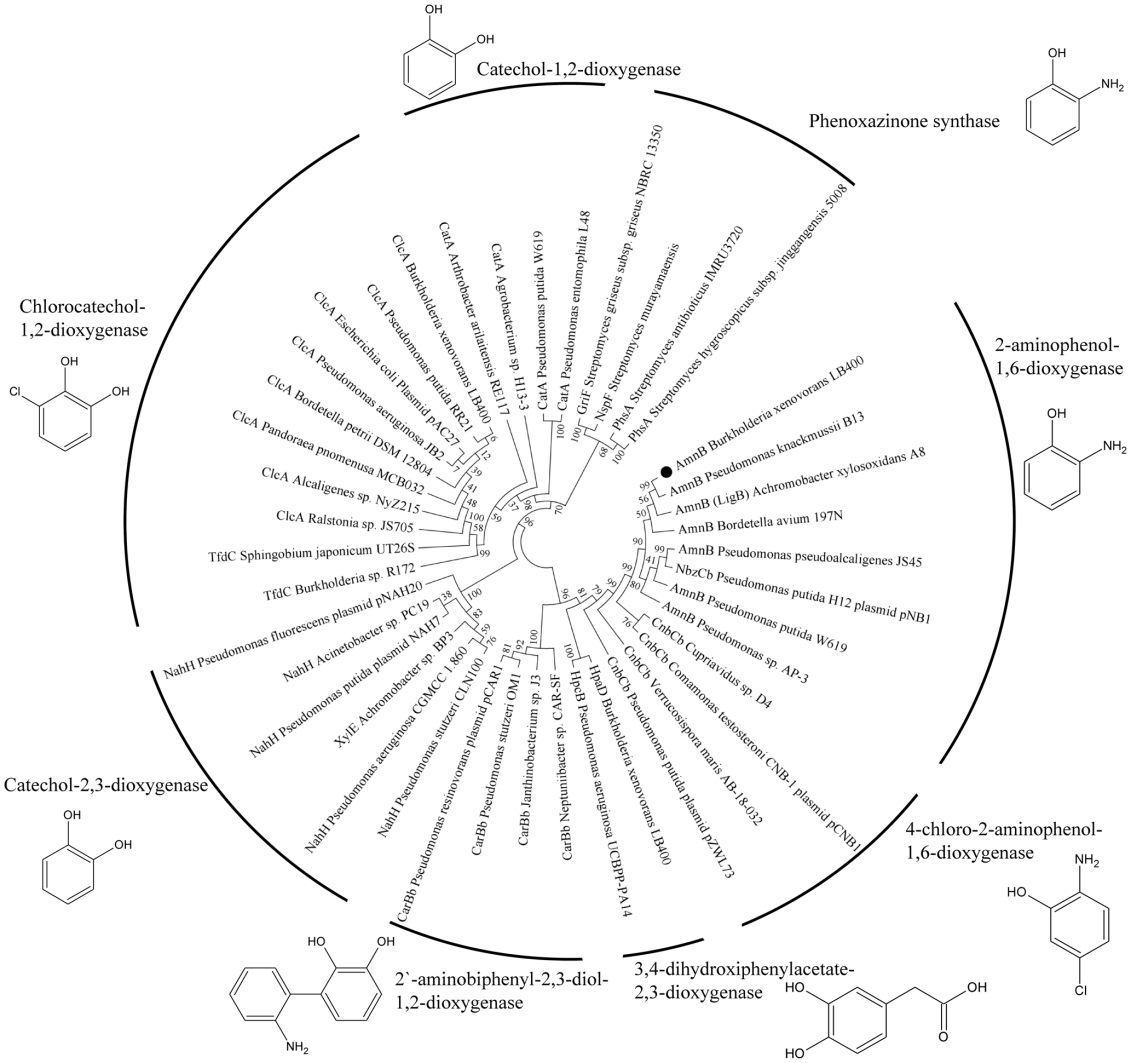
Phylogenetic tree showing the relatedness of AmnB and other class III ring-cleavage dioxygenases. The dendrogram was constructed by the neighbor-joining method using MEGA 5.02 based on sequence alignments calculated by Clustal W. Sequence of deduced protein from *amnB* gene from *B. xenovorans* strain LB400 is highlighted (black circle). The proteins (accession number) are: AmnB *B. xenovorans* LB400 (YP559855.1); AmnB *P. knackmussii* B13 (CAE92875.1); AmnB (LigB) *Achromobacter xylosoxidans* A8 (YP003978676.1); AmnB *Bordetella avium* 197N (YP785083.1); AmnB *P. pseudoalcaligenes* JS45 (AAB71524.1); NbzCb *P. putida* HS12 plasmid pNB1 (AAK26519.1); AmnB *P. putida* W619 (YP001748883.1); AmnB *Pseudomonas* sp. AP-3 (O33477.1); CnbCb *C. testosteroni* CNB-1 plasmid pCNB1 (YP001967698.1); CnbCb *Cupriavidus* sp. D4 (AEI74584.1); CnbCb *Verrucosispora maris* AB-18-032 (YP004405267.1); CnbCb *P. putida* plasmid pZWL73 (DQ306889.1); HpaD *B. xenovorans* LB400 (YP553304.1); HpcB *P. aeruginosa* UCBPP-PA14 (ABJ13394.1); CarBb *Neptuniibacter* sp. CAR-SF (BAG30828.1); CarBb *Janthinobacterium* sp. J3 (BAC56744.1); CarBb *P. stutzeri* OM1 (BAA31269.1); CarBb *P. resinovorans* plasmid pCAR1 (BAC41547.1); NahH *Acinetobacter* sp. PC19 (AAW81679.1); NahH *P. putida* plasmid NAH7 (AAA98183.1); XylE *Achromobacter* sp. BP3 (ACF20629.1); NahH *P. stutzeri* CLN100 (CAD62376.1); NahH *P. aeruginosa* CGMCC 1.860 (ACV05014.1); NahH *P. fluorescens* plasmid pNAH20 (AAW81680.2); TfdC *Sphingobium japonicum* UT26S (YP_003544400.1); TfdC *Burkholderia* sp. R172 (Q7WYF8); ClcA *Ralstonia* sp. JS705 (CAA06968.1); ClcA *Alcaligenes* sp. NyZ215 (ABQ23434.1); ClcA *Pandoraea pnomenusa* MCB032 (ABS81346.1); ClcA *B. xenovorans* LB400 (ABE31816.1); ClcA *E. coli* plasmid pAC27 (AAA98281.1); ClcA *P. aeruginosa* JB2 (AAC69474.1); ClcA *P. putida* RR21 (CAE92861); ClcA *Bordetella petrii* DSM12804 (CAP41873.1); CatA *Arthrobacter arilaitensis* Re117 (YP_003918477.1); CatA *P. putida* W619 (ACA73195.1); CatA *P. entomophila* L48 (CAK15903.1); CatA *Agrobacterium* sp. H13-3 (YP_004279743.1); PhsA *Streptomyces hygroscopicus* subsp. *jinggangensis* 5008 (YP006248289.1); PhsA *Streptomyces antibioticus* IMRU3720 (AAA86668.1); GriF *Streptomyces griseus* subsp. *griseus* NBRC13350 (BAG21075.1); NspF *Streptomyces murayamaensis* (BAJ08174.1).

The phylogenetic sequence analysis of the 2-aminophenol 1,6-dioxygenase AmnB β-subunits and related class III ring-cleavage dioxygenases is illustrated in Figure 3. The 2-aminophenol 1,6-dioxygenase β-subunits were clustered in two branches. One branch includes the AmnB proteins of *B. xenovorans* LB400, *P. knackmussii* B13, *B. avium* 197N and *Achromobacter xylosoxidans* A8. The second branch comprises 2-aminophenol 1,6-dioxygenase β-subunits from *Pseudomonas* sp. AP-3, *P. putida* W619, *P. putida* HS12 and *P. pseudoalcaligenes* JS45. This sequence analyses suggest that AmnB protein of *B. xenovorans* LB400 evolved separately from AmnB protein of *Pseudomonas* sp AP-3 and *P. pseudoalcaligenes* strain JS45. This is in accordance with the genetic organization of *amn* gene clusters (Fig. 2). The gene organization of *amnJRBACD* cluster of *B. xenovorans* strain LB400, *P. knackmussii* B13, *B. avium* 197N is identical, and their AmnB enzymes were classified in the same branch in the phylogenetic tree. *P. pseudoalcaligenes* JS45 and *Pseudomonas* sp AP-3 showed a different *amn* gene cluster than *B. xenovorans* strain LB400 and also their AmnB proteins were classified in a different phylogenetic branch. The AmnB proteins are closely related to the 2-amino-5-chlorophenol-1,6-dioxygenase CnbCb proteins that oxidizes 4-chloro-2-aminophenol, but less related to dioxygenases subunits involved in the oxidation of 3,4-dihydroxyphenylacetate and 2′-aminobiphenyl-2,3-diol. All these dioxygenases shared a common ancestor with catechol 2,3-dioxygenase NahH and XylE proteins. Phenoxazinone synthases, which use the same substrate as AmnB proteins, are closely related to catechol 1,2-dioxygenases but not related to 2-aminophenol 1,6-dioxygenase AmnB β-subunits.

### Transcription of the *amn* Genes

**Figure 4:**
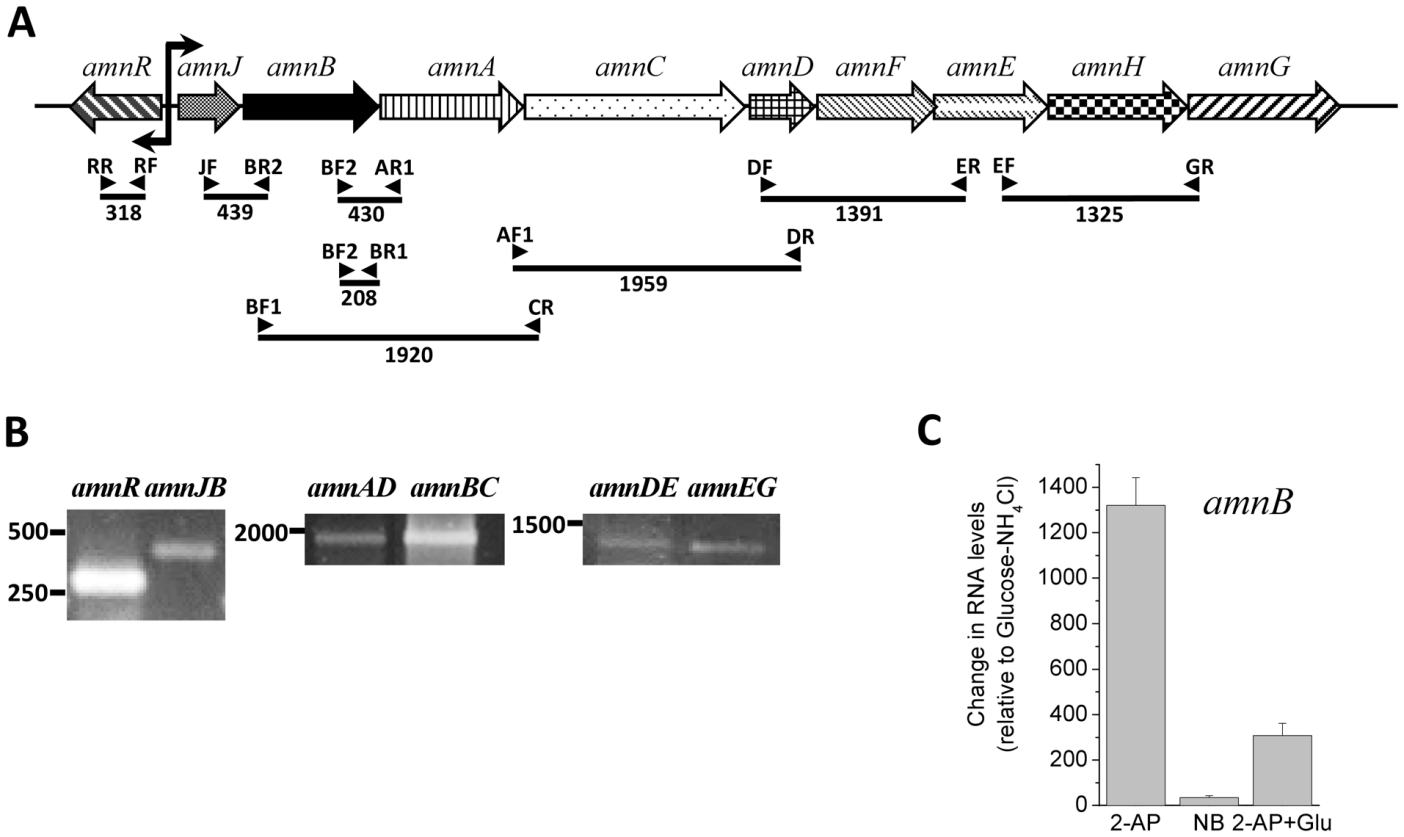
Transcriptional analysis of the *amn* genes. A, Organization of the *amn* genes and schematic representation of the location and size of the amplicons obtained by RT-PCR experiments. Predicted promoters are shown as break arrows, bent in the directions of transcription. Primers are represented by arrowheads. B, PCR products from RT-PCR experiments using as templates mRNA obtained from LB400 cells collected at early exponential growth phase in liquid BLKN minimal medium with glucose (10 mM) and 2-AP (1 mM) as sole carbon and nitrogen source, respectively. The primer pairs used are listed in Table 1. The amplification products are *amnR* (318 bp), *amnJB* (439 bp), *amnACD* (1959 bp), *amnBAC* (1920 bp), *amnDFE* (1391 bp) and *amnEHG* (1325 bp). C, Transcriptional analysis of the *amnB* gene. RT-qPCR assays were performed using mRNA from LB400 cells grown in BLKN minimal medium supplemented with glucose (10 mM) and NH_4_Cl (1 mM) until exponential phase (turbidity at 600 nm of 0.5) and further incubated for 1 h in BLKN medium in presence of glucose (10 mM) and NH_4_Cl (1 mM), 2-aminophenol (1 mM) (2-AP), nitrobenzene (1 mM) (NB) or glucose (10 mM) and 2-AP (1 mM) (Glu+2-AP). The primer pairs used are listed in Table 1. The *ftsZ* gene was used as a reference gene.

The transcription of the *amn* genes in strain LB400 was studied using specific primer sets (Fig. 4A). To study the co-expression of the *amn* genes, diverse primers sets were designed to amplify adjacent genes. RT-PCR of mRNA from LB400 cells grown until exponential phase in BLKN medium with glucose and 2-AP as sole carbon and nitrogen sources were performed. The expression of the *amnR* gene in strain LB400 was observed (Fig. 4B). Two divergent σ^70^-type promoters were identified in the *amn* gene cluster, which are located between the *amnR* gene and the *amnJ* gene. RT-PCR analyses showed the co-expression for *amnJB*, *amnBAC*, *amnACD*, *amnDFE* and *amnEHG* genes (Fig. 4B), indicating that the *amn* gene cluster is an operon in strain LB400. The co-expression of the *amn* genes strongly suggests that the promoter located upstream of the *amnJ* gene permits the expression of an *amnJBACDFEHG* single transcript. A putative MarR-type regulatory binding sequence (TAGTACGTATACGTTTCA) was identified upstream of the *amnJBACDFEHG* gene cluster, which showed a consensus MarR-type regulator binding sequence (5′-TAG(G/T)ACGTATACGTACTA-3′) located upstream of the *amnJ* gene from diverse 2-AP-degrading bacteria: *P. putida* W619, *C. testosteroni* CNB1, *P. pseudoalcaligenes* JS45, *P. knackmussii* B13 and *Bordetella avium* 197N. The *amnB* gene encodes the 2-aminophenol 1,6-dioxygenase beta subunit, which is the key enzyme of 2-AP catabolic pathway. The expression of *amnB* gene in LB400 cells grown in glucose in presence or absence of 2-AP and nitrobenzene was studied. Real time RT-PCR analysis showed that the *amnB* gene has a basal expression during growth in glucose. The *amnB* gene expression was highly induced by 2-AP, whereas lower induction was observed in cells during exposure to 2-AP and glucose (Fig. 4C). The lowest induction of *amnB* gene expression was observed in cells during exposure to nitrobenzene. Overall these results indicate a regulated expression of the *amn* gene cluster in strain LB400.

### 2-aminophenol Degradation

**Figure 5:**
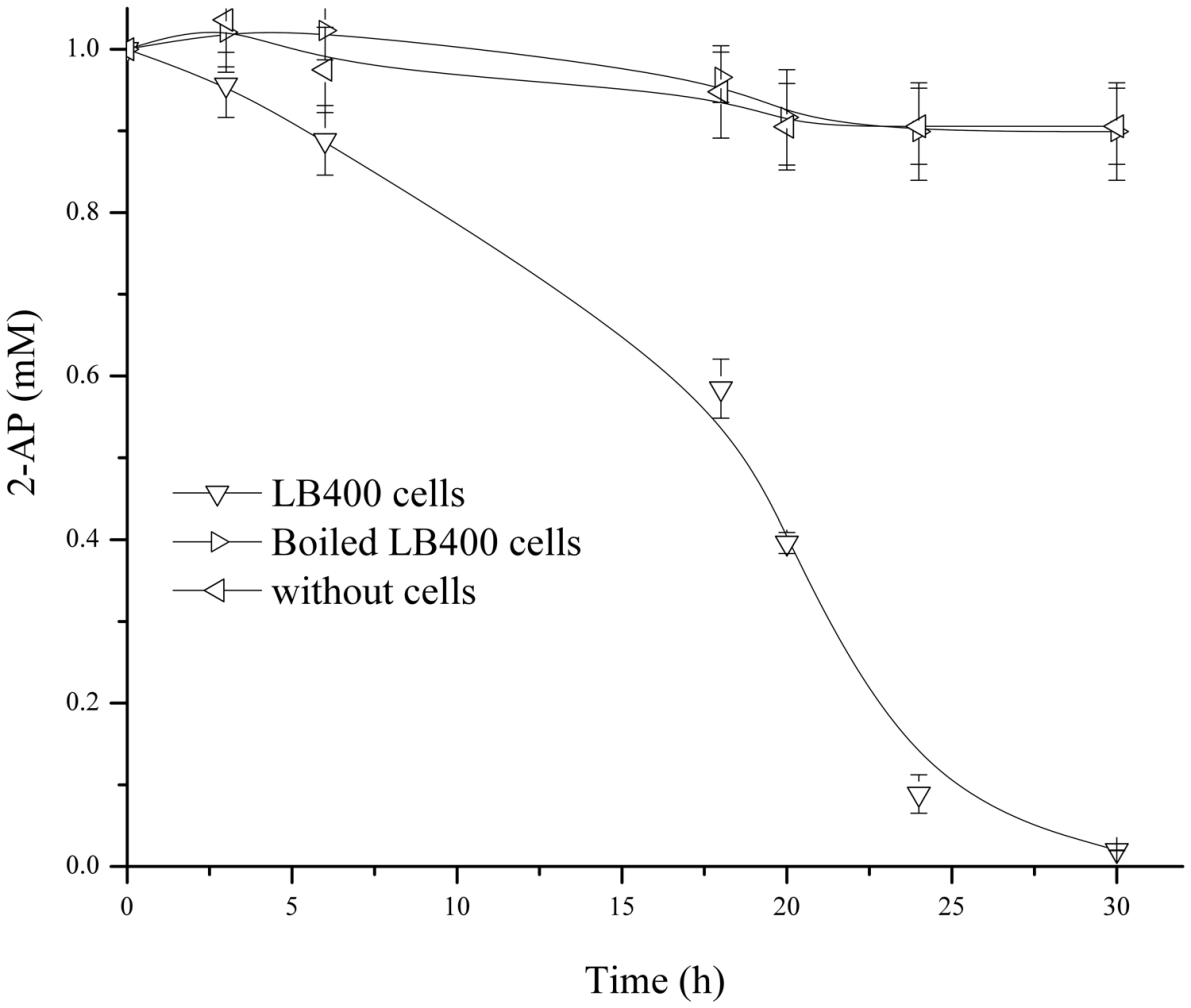
2-aminophenol degradation by *B. xenovorans* LB400. Glucose-grown LB400 cells were incubated in phosphate buffer with 2-AP (1 mM). Control assays with boiled cells or without cells are depicted. Each point is an average ± SDs of results from, at least, three independent assays.

To further characterize the 2-AP catabolic pathway, 2-aminophenol degradation by strain LB400 was studied. Resting LB400 cells were incubated with 2-AP. LB400 cells degrade 15% of 2-AP after 6 h of incubation, whereas complete degradation was observed after 30 h (Fig. 5). In contrast, 2-AP was not degraded by boiled LB400 cells. The *amnBA* ^−^ mutant of strain LB400 is not able to degrade 2-AP, indicating that 2-aminophenol-1,6-dioxygenase is the sole enzyme involved in 2-AP degradation in strain LB400.

### Growth of Strain LB400 on 2-aminophenol

The expression of the *amn* genes and the degradation of 2-AP, suggest a functional 2-AP catabolic pathway in *B. xenovorans* strain LB400. In a further analysis of this metabolic pathway, the growth of strain LB400 in minimal medium with 2-AP as nitrogen or carbon source was studied. In a first approach, the growth of strain LB400 on BLKN agar plates in presence of 2-aminophenol crystals in absence or presence of glucose was studied. Small colonies of LB400 cells grown around the 2-AP crystals used as sole nitrogen source or as sole carbon and nitrogen source on BLKN plates were observed (data not shown). An increased number of small colonies around the 2-AP crystals on plates with 2-AP and supplemented with nitrobenzene was observed, suggesting that nitrobenzene stimulate growth probably by the induction of the 2-AP catabolic pathway genes. These small colonies were phenotypically confirmed as *B. xenovorans* LB400. The bacteria of the colonies were able to grow in minimal medium supplemented with biphenyl as sole carbon and energy source and show typical colonies on OFBPL medium agar (data not shown). In contrast, no colonies were observed on plates with nitrobenzene as sole carbon and nitrogen source (data not shown).

**Figure 6:**
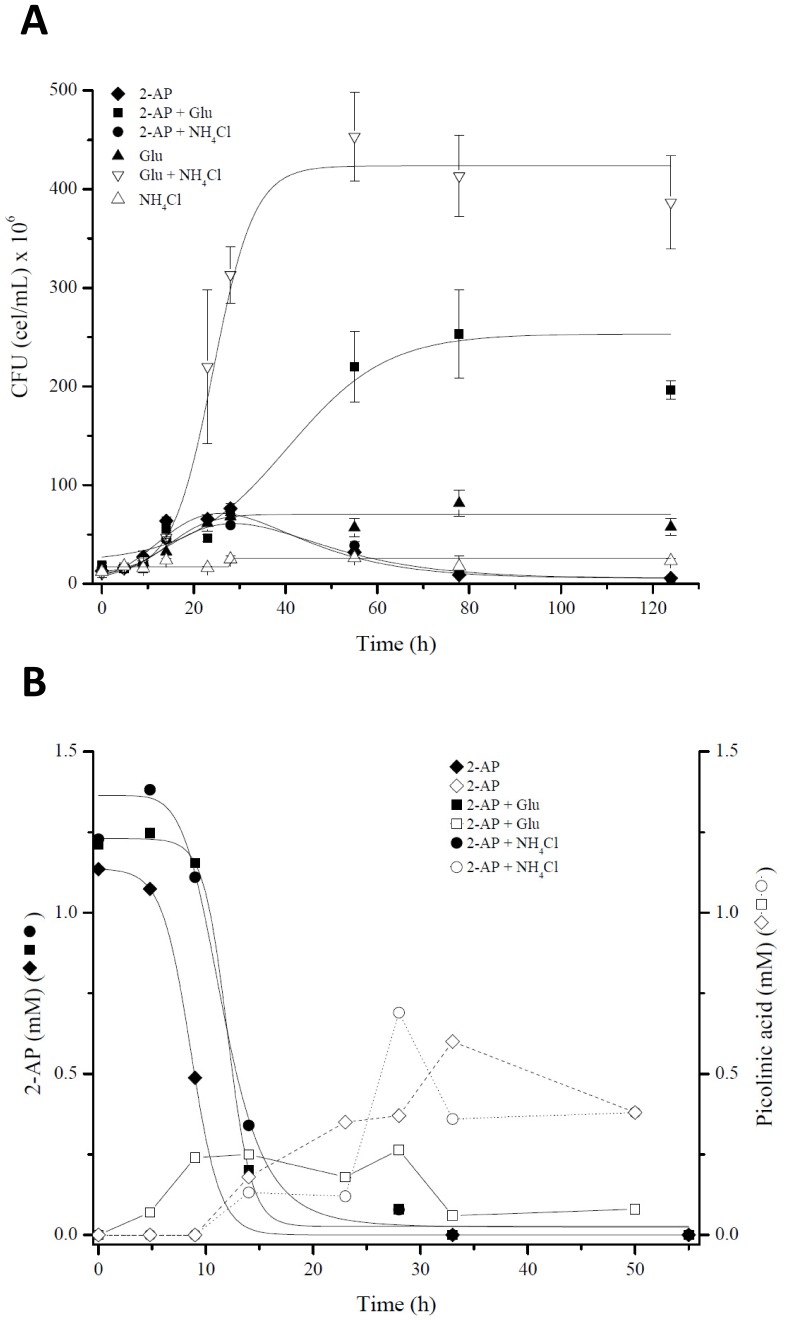
Growth of strain LB400 on 2-aminophenol. A, Growth curve of strain LB400 in liquid BLKN minimal medium supplemented with 2-AP (1 mM), glucose (10 mM) and NH_4_Cl (1 mM). Growth was monitored measuring CFU. Each point is an average ± SDs of results from, at least, three independent assays. B, Degradation of 2-AP (filled symbols) and production of picolinic acid (empty symbols) during LB400 growth under these conditions were quantified by HPLC.

In a second approach, the growth of strain LB400 in liquid BLKN medium with 2-AP (1 mM) as sole nitrogen source or as sole nitrogen and carbon source was analyzed. Growth of LB400 cells in BLKN medium with 2-AP as sole nitrogen source and glucose as carbon source was observed (Fig. 6A). However, the cells reached lower biomass than cells incubated with NH_4_Cl as sole nitrogen source and glucose as sole carbon source. These results suggest that an inhibitory metabolite was produced during LB400 growth on 2-AP and glucose. LB400 cells showed a minimal growth using 2-AP as sole carbon and nitrogen source, which was similar to growth on glucose in absence of a nitrogen source, suggesting that in absence of other carbon and nitrogen source, 2-AP could be used as carbon source, but not as nitrogen source. No LB400 growth was observed in presence of NH_4_Cl and absence of a carbon source (Fig. 6A). To link the LB400 growth on 2-AP with the *amn*-encoded catabolic pathway, the growth on 2-AP of an *amnBA* ^−^ mutant derivative of *B. xenovorans* LB400 generated by homologous recombination was studied. The *amnBA* ^−^ mutant was unable to grow in BLKN medium with 2-AP as sole nitrogen source and glucose as carbon source, which correlates with no degradation of 2-AP (data not shown).

To study if during the degradation of 2-AP by strain LB400 a toxic metabolite was produced, the supernatants of cultures mentioned above were analyzed by HPLC. 2-AP was degraded during the first 15 h, independently if 2-AP was used as nitrogen or as carbon source (Fig. 6B). Interestingly, 2-AP degradation correlates with the production of a metabolite. This metabolite was identified as picolinic acid (PA) by comparison of its absorption spectrum and HPLC retention time with an authentic standard. Picolinic acid could be produced by a spontaneous non-enzymatic reaction than convert 2-AMS into PA. 2-AMS is produced from 2-AP by the 2-aminophenol-1,6-dioxygenase. During 2-AP degradation only partial conversion into PA was observed. The maximal PA concentration reached during growth using 2-AP (1 mM) as carbon source or as carbon and nitrogen source was approximately 0.5 mM, which correlates with a low cell growth. When 2-AP was used as sole nitrogen source a higher biomass was reached whereas a lower PA concentration (0.25 mM) was observed.

**Figure 7:**
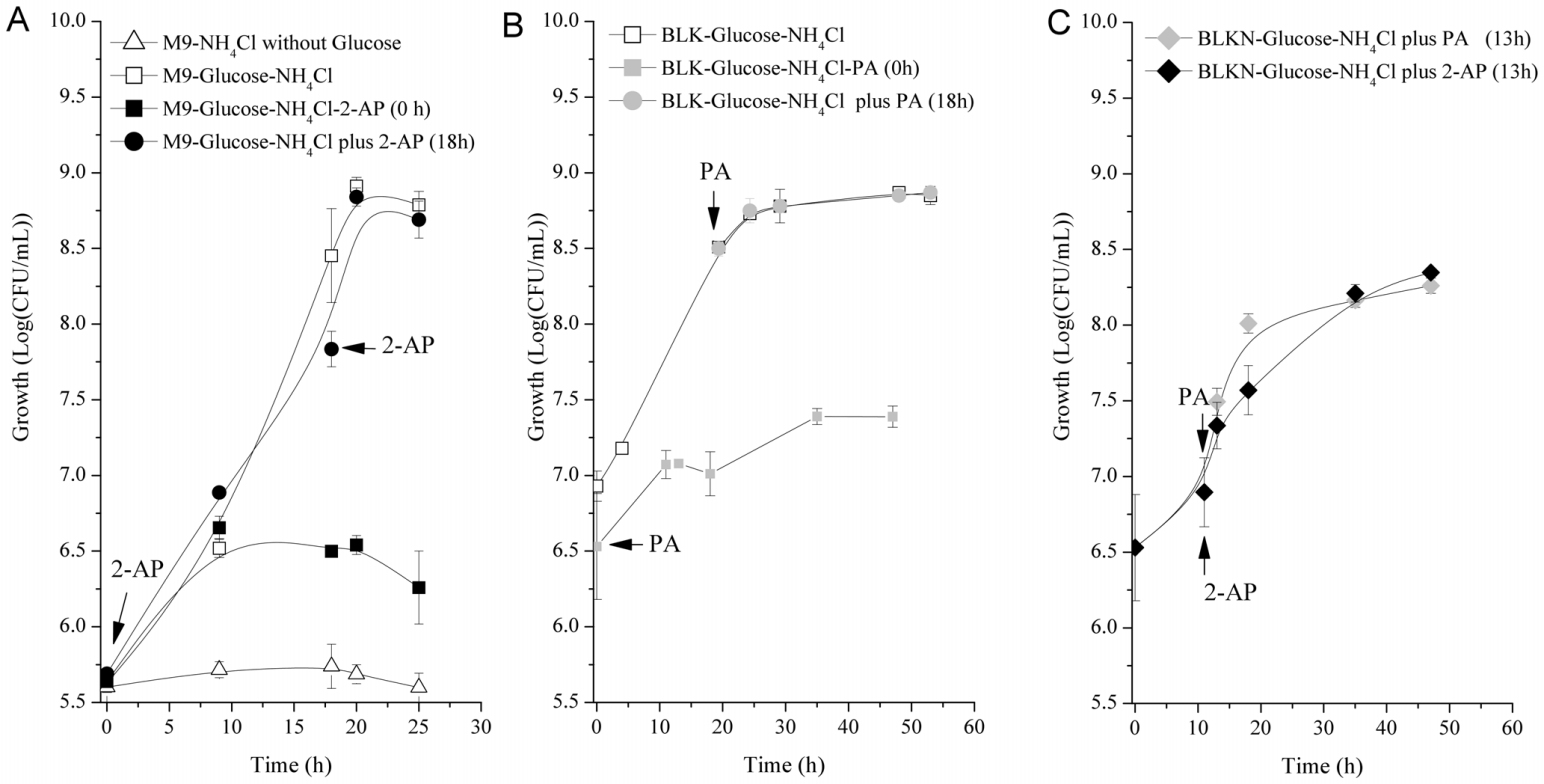
Effects of 2-aminophenol and picolinic acid on *B. xenovorans* LB400 growth. A, LB400 cells were cultured in M9 minimal medium with glucose (10 mM) as sole carbon source and NH_4_Cl (1 mM) as sole nitrogen source. Two cultures were supplemented with 2-AP (1 mM) at time 0 or at late exponential phase (18 h). As controls, LB400 cells were cultured in M9 minimal medium without a carbon source or cultured with glucose. B, LB400 cells were cultured in BLKN minimal medium with glucose (10 mM) and NH_4_Cl (1 mM) as sole carbon and nitrogen source, respectively. Two cultures were supplemented with picolinic acid (1 mM) at time 0 or at late exponential phase (18 h). C, LB400 cells cultured in BLKN minimal medium with glucose (10 mM) and NH_4_Cl (1 mM), were supplemented after 13 h with 2-AP (1 mM) or PA (1 mM).

Figure 7 illustrate the effects of 2-AP and PA on *B. xenovorans* strain LB400 growth. LB400 growth on glucose and NH_4_Cl was not affected by the addition of 2-AP at late exponential phase (Fig. 7A). In contrast, when 2-AP was present since lag phase, a low cell density was reached at stationary phase. These results suggest that 2-AP has to be metabolized to exert an inhibitory effect on LB400 growth. On the other side, the effect of the addition of PA on LB400 growth was studied (Fig. 7B). The addition of PA at the lag phase reduced significantly the biomass reached at exponential phase of LB400 culture growing with glucose and NH_4_Cl. However, the addition of PA at late exponential phase did not affect the cell growth. The addition of 2-AP or PA at mid-exponential phase of LB400 cultures growing with glucose and NH_4_Cl inhibited also the cell growth (Fig. 7C). Finally, the minimal inhibitory concentration (MIC) of PA for *B. xenovorans* strain LB400 was measured. The MIC of PA that prevented LB400 growth was 2 mM.

## Discussion

This study revealed that the 2-aminophenol catabolic central pathway is functional in the model aromatic-degrading bacterium *B. xenovorans* LB400. Strain LB400 is able to use 2-AP as sole nitrogen source for growth, indicating an active 2-AP catabolic central pathway. Only few bacterial strains capable to grow on 2-AP have yet been reported [5], [6]. *Pseudomonas* sp. strain AP-3 is able to grow in liquid medium using 2-AP as sole carbon and nitrogen source [5]. *P. pseudoalcaligens* strain JS45 grow in liquid medium using 2-AP as sole carbon and nitrogen source but only when the cells were previously induced with NB [6]. In contrast, the nitrobenzene-degrader *Pseudomonas putida* strain HS12 did not grow on 2-AP [7]. Interestingly, *P. knackmussi* strain B13 that share a highly identical *amn* gene cluster with *B. xenovorans* LB400 is not able to grow on 2-AP [9]. In our study, strain LB400 reached lower cell numbers in liquid medium using as sole nitrogen source for growth 2-AP (2.5×10^8^ CFU mL^−1^) than ammonium chloride (4.5×10^8^ CFU mL^−1^). On the other side, only minimal growth of strain LB400 was measured in liquid medium with 2-AP as sole nitrogen and carbon source. In addition, small colonies of strain LB400 were observed on agar plates using 2-AP crystals as sole nitrogen and carbon source. All these results indicate a reduced growth of strain LB400 on 2-AP, suggesting an inhibition of cell growth during the metabolism of 2-AP. The growth inhibition suggests the production of a toxic metabolite. In this study, we were able to demonstrate that during the degradation of 2-AP by strain LB400 this substrate is partially converted into the antibiotic picolinic acid. When 2-AP (1 mM) was used as sole carbon and nitrogen source for strain LB400 growth, high levels of PA (0.5 mM) were produced. In this report we showed that PA inhibited the growth of *B. xenovorans* strain LB400. The MIC of PA for strain LB400 is 2 mM. PA is a well known antibiotic for Gram-negative bacteria such as *P. aeruginosa* and *Staphylococcus aureus*, *Mycobacterium avium* complex and fungi such as *Candida albicans* [31]–[33]. PA is a chelating agent for metals ions such as Fe^2+^ and Zn^2+^. Interestingly, it has been reported that PA induces antitumoral activity of macrophages and chromium picolinate has been used as a dietary supplement for obese persons to promote weight loss [31]–[36]. Nishino and Spain [6] reported that crude extracts of *P. pseudoalcaligenes* strain JS45 transformed 2-AP through 2-AMS into PA. The addition of NAD to the extracts of strain JS45 prevented the formation of PA by favoring the formation of 2-aminomuconate by the 2-aminomuconic-6-semialdehide dehydrogenase AmnC. For the production of PA, a recombinant *E. coli* strain harboring only the genes for 2-aminophenol 1,6-dioxygenase from strain JS45 was used for the biotransformation of 2-AP into PA [37]. *P. knackmussi* strain B13 that has a highly identical 2-AP catabolic pathway as strain LB400 did not grow on 2-AP [9]. To explain the failure of strain B13 growth on 2-AP, Gaillard *et al.* proposed the production of a toxic intermediate from 2-AP or alternatively a metabolic misrouting. Our study demonstrates the conversion of 2-AP into PA by the *amn*-encoded catabolic pathway in *B. xenovorans* strain LB400. These results suggest that strain B13 also produces the toxic PA during 2-AP degradation, impeding its growth on 2-AP. The biotransformation of aromatic compounds into toxic metabolites and antibiotics has been reported [38]–[41]. Cámara *et al.* [40] reported that PCBs were converted by enzymes of the biphenyl catabolic pathway into highly toxic biphenyl dihydrodiols and dihydroxylated biphenyls. Blasco *et al.* [38], [39] demonstrated that chlorobenzoates were metabolized by bacteria through 4-chlorocatechol into the antibiotic protoanemonin.

Genome analysis indicates that the *amn* genes for 2-aminophenol degradation are clustered in the *clc* genomic island at C1 from *B. xenovorans* LB400. LB400 *clc* genomic island shows 99% sequence identity with the *clc* element of *P. knackmussii* strain B13 and contains genes of the 2-aminophenol and chlorocatechol catabolic pathways and genes involved in the transport of aromatic compounds. The *amn* gene clusters from *B. xenovorans* LB400 and *P. knackmussii* strain B13 have the same *amn* gene organization and the *amn* genes of both strains have almost identical gene sequence [9], which strongly suggest horizontal gene transfer of the *amn* gene cluster between bacteria. Genome analysis of strain LB400 indicates the absence of *nbz* (*hab*) genes involved in the conversion of nitrobenzene into 2-aminophenol. In accordance with the genome analysis, strain LB400 was unable to grow using nitrobenzene as sole nitrogen or carbon source. In contrast, both 2-AP-degrading *Pseudomonas* strains JS45 and HS12 possess the *nbzA* (*habA*) and *nbzB* (*habB*) genes for nitrobenzene degradation and are able to grown on nitrobenzene as carbon and nitrogen energy source [6], [42].

The 2-aminophenol-1,6-dioxygenase AmnB subunit of *B. xenovorans* LB400 is closely related to AmnB of *P. knackmussi* strain B13 (100% identity) and LigB of *B. avium* 197N (89% identity). 2-aminophenol-1,6-dioxygenase belong to the extradiol dioxygenases [8], [43] that include also the 2-amino-5-chlorophenol-1,6-dioxigenase and the catechol-2,3-dioxygenase. The substrates of these related extradiol dioxygenases comprise catechol, 2′-aminobiphenyl-2,3-diol, 3,4-dihydroxybiphenylacetate, 2-amino-5-chlorophenol and 2-aminophenol, suggesting the evolution of these catabolic enzymes. The extradiol dioxygenases are classified in two branches. The enzymes of substrates with a catechol group such as 2′-aminobiphenyl-2,3-diol 1,2-dioxygenases and 3,4-dihydroxyphenylacetate 2,3-dioxygenases are closely related to catechol 2,3-dioxygenases. The extradiol dioxygenases that recognize substrate in which one hydroxyl group of the catechol moiety has been replaced by an amino group are most distantly related to the catechol 2,3-dioxygenases. The 2-aminophenol-1,6-dioxigenase AmnB of *Pseudomonas* sp. strain AP3 is closely related to 2-amino-5-chlorophenol-1,6-dioxigenase CnbCb subunit of *C. testosteroni* strain CNB-1 (70% identity). In addition, the organization of the *cnb* gene cluster of *C. testosteroni* CNB-1 and the *amn* gene cluster of *P. putida* HS12 is conserved, suggesting a common origin. The phenoxazinone synthase (GriF, PhsA or NspF) of several members of the *Streptomyces* genus that use also 2-AP as substrate are not related to 2-aminophenol-1,6-dioxygenase [44]. This is not surprising since phenoxazinone oxidase is not an extradiol dioxygenase.

In this study, we showed that *B. xenovorans* strain LB400 grew using 2-AP as sole nitrogen source and that LB400 cells degraded completely 2-AP. In contrast, an *amnBA* ^−^ mutant was unable to grow on 2-AP as nitrogen source and to degrade this compound, linking the *amn*-encoded central pathway with 2-AP catabolism in strain LB400. In addition, RT-PCR analysis showed that *amnA*, *amnB*, *amnC*, *amnD, amnE, amnF*, *amnG, amnH, amnJ* and *amnR* genes were expressed in LB400 cells. These results demonstrate that the *amn* gene cluster is functional. The co-expression of the *amnJB*, *amnBAC*, *amnACD*, *amnDFE* and *amnEHG* genes and the presence of a promoter upstream of the *amnJ* gene indicate that the *amn* genes are clustered in an operon. The expression of the *amnB* gene in LB400 cells grown in glucose indicates a basal expression of the *amn* gene. The *amnB* gene expression is highly induced by 2-AP, whereas lower induction was observed in presence of 2-AP and glucose. These results suggest an induction of the *amnB* gene transcription by 2-AP or its metabolic intermediates and a down regulation of its transcription by glucose. In accordance with the regulation of the *amn* genes observed in our study, transcriptional analysis with a DNA chip showed a higher expression of the *amn* genes of strain LB400 in benzoate than in succinate [45]. The predicted *amnR* gene-encoded MarR-type transcriptional regulator of strain LB400 is probably a transcriptional repressor of the *amn* gene cluster. MarR-type transcriptional regulators bind as dimmers to palindromic sequences within the promoter. A regulatory palindromic sequence was observed in the intergenic region of the *amnR* gene and the *amnJBACDFEHG* gene cluster from strain LB400. MarR regulators bind to DNA in absence of the specific ligand, causing generally transcriptional repression [46]. *P. knackmussi* strain B13 showed by dot blot hybridization a basal *amnB* gene expression in glucose and a 50-fold increase *amnB* gene expression in 2-AP (1 mM) and anthranilate (1 mM) [9]. *P. putida* HS12 down regulated the *amn* genes in succinate [7]. In contrast, *Pseudomonas* sp. strain AP3 expresses constitutively the *amn* genes [8], which correlates with the absence of the *amnR* gene in its *amn* gene cluster.

Figure 1 illustrates the predicted functional 2-AP catabolic pathway of the model bacterium *B. xenovorans* strain LB400. 2-Aminophenol-1,6-dioxygenase encoded by the *amnBA* genes meta-cleaved 2-aminophenol into 2-AMS. The *amnBA* ^−^ mutant is not able to degrade 2-AP, indicating that 2-aminophenol-1,6-dioxygenase is the sole enzyme involved in 2-AP degradation in strain LB400. 2-AMS is further oxidized by 2-aminomuconic-6-semialdehyde dehydrogenase AmnC into 2-AM. Alternatively, 2-AMS could chemically rearrange into PA (Fig. 1). The 2-AM is deaminated by 2-aminomuconic deaminase AmnD releasing 4-oxalocrotonic acid and ammonium. The ammonium released at this step could be used as nitrogen source for bacterial growth. 4-oxalocrotonate is converted into pyruvate, acetaldehyde and acetil-CoA, by catabolic enzymes that are also involved in the catechol metabolism.

In conclusion, this study indicates that *B. xenovorans* strain LB400 possess a functional 2-AP catabolic pathway, which channel the aromatic pollutant 2-AP into the Krebs cycle. Interestingly, the 2-AP degradation in strain LB400 could lead to the production of the antibiotic picolinic acid, which inhibits its growth. The versatile catabolic repertoire for aromatic compounds of *B. xenovorans* LB400 allows the degradation of aromatic pollutants with chloro or amino substituent’s such as PCBs and 2-AP, which could be useful for bioremediation studies.
